# Nurses' Experiences of Psychological Safety in Ad Hoc Teams During Emergency Care: An Interview Study

**DOI:** 10.1111/jan.70413

**Published:** 2025-12-07

**Authors:** Jenny Stenvall, Samuel Edelbring

**Affiliations:** ^1^ Örebro University Örebro Sweden; ^2^ Mälardalens University Västerås Sweden

**Keywords:** ad hoc teams, collaboration, emergency care, leadership, nurse, nursing, organisational culture, psychological safety, team dynamics, trust

## Abstract

**Aim:**

To investigate specialist nurses' experience of psychological safety in ad hoc teams during emergency care.

**Design:**

Interpretive descriptive qualitative study.

**Methods:**

Semi‐structured interviews with nine specialist nurses were conducted in Sweden from May to June 2024 and analysed using reflexive thematic analysis (Braun & Clarke).

**Results:**

Four themes were identified: Interpersonal skills: implications for psychological safety; Individuality and team dynamics; Confidence, competence and collaboration; and Organisational responsibility for promoting psychological safety.

**Conclusion:**

Psychological safety in ad hoc emergency care teams is a fragile and multifaceted phenomenon, shaped by interpersonal skills, leadership and organisational culture. Supportive environments characterised by open communication and proactive leadership enable specialist nurses to collaborate confidently and safely, even under acute stress. Targeted efforts to strengthen these factors are essential for optimising teamwork and patient outcomes.

**Implications for the Profession:**

The psychological safety implications for specialist nurses in ad hoc teams during emergency care are profound. Psychological safety fosters an environment that empowers nurses to leverage clinical expertise, collaborate in ad hoc teams and improve patient outcomes. Promoting psychological safety ensures specialist nurses feel respected, valued and secure, leading to better care and a more resilient workforce.

**Impact:**

This qualitative study investigated specialist nurses' experience of psychological safety in ad hoc teams in acute care. The results will influence the awareness of nurses, specialist nurses, other professions, managers and organisations about the importance of feeling psychologically safe.

**Reporting Method:**

Presentation follows COREQ 32‐item checklist.

**Patient and Public Involvement:**

No patient or public involvement.

**Contribution to Global Community:**

Shows that psychological safety helps nurses perform in ad hoc emergency teams. Identifies key factors that affect teamwork and patient care.

## 1 Introduction

Effective teamwork among experienced nurses is fundamentally supported by interpersonal skills, with psychological safety playing a central role. Psychological safety, as defined by Rahmati and Poormirzaei (2018), involves responding constructively to interpersonal conflicts, not only recognising them. The consequences of psychological safety become particularly crucial in high‐pressure environments such as emergency care, where nurses frequently work in ad hoc teams urgently formed to address acute and often complex patient needs. In these settings, rapid trust and cohesion are essential for effective decision‐making. However, research indicates that these ad hoc teams, often lacking pre‐established relationships, face significant challenges regarding psychological safety—an essential component for optimal communication and collaboration (Bahadurzada et al. 2024). Therefore, it is crucial to understand how psychological safety in ad hoc teams in these dynamic, high‐stakes environments is experienced by experienced nurses.

Psychological safety has been established as a critical factor for effective collaboration and organisational learning in a global context. In multicultural and cross‐functional teams, it fosters open communication, inclusion and innovation, particularly in organisations characterised by rapid change and knowledge‐intensive processes (Edmondson 1999; Edmondson and Lei 2014). In healthcare, the team function is crucial, especially critical in high‐stakes settings like acute emergency care, where experienced nurses face intense pressure that demands rapid decision‐making, precision and expertise. In such environments, psychological safety enables professionals to work confidently and communicate openly, ultimately enhancing performance and patient outcome (Murray et al. 2022; Shankar et al. 2025).

## 2 Background

The concept of *psychological safety*, first introduced by Schein and Bennis (1965) and later refined by Edmondson (1999, 2019), has become a foundational construct for understanding team dynamics in healthcare settings. Edmondson defines psychological safety as “a belief that one will not be penalized or humiliated for speaking up with ideas, questions, concerns or mistakes,” underscoring its critical role in fostering open communication and supporting organisational learning. Central to Edmondson's perspective is the importance of group‐level interactions and the social norms constructed through shared experience, rather than individual perception alone. Edmondson further emphasises how psychological safety develops through team interactions and shared team beliefs, rather than just taking individual attributes into consideration.

In emergency care, where rapid decision‐making and effective teamwork are crucial, psychological safety is particularly important. Recent studies highlight its significance in enhancing patient safety, optimising team performance and supporting quality improvement initiatives. For example, Purdy et al. (2023) found a positive correlation between psychological safety in emergency departments and improved team performance and patient safety outcomes. Similarly, staff who perceive a psychologically safe environment are more likely to engage in open communication, report errors and participate in quality improvement work (Ren et al. 2022; Cho et al. 2023).

Effective teamwork is essential for ensuring patient safety and timely interventions (White et al. 2018, 2025). Nurses, in particular, frequently work within ad hoc teams—rapidly assembled groups of healthcare professionals who may have little or no prior experience working together. These ad hoc teams often operate in high‐pressure and complex environments, which demand swift coordination and clear role allocation to maintain patient safety.

These teams are common in high‐stakes environments such as emergency departments, trauma units and intensive care units, where swift coordination and role clarity are critical. However, their transient nature presents unique challenges; limited familiarity among members often hinders trust‐building, effective role allocation and seamless collaboration (Mannion and Thompson 2014; White et al. 2025). Prior research suggests that fostering psychological safety can help mitigate these challenges by promoting trust and collaboration (Edmondson 1999; O'Donovan and McAuliffe 2020), thereby providing a rationale for exploring this concept in the context of ad hoc nursing teams.

An open culture in which mistakes are viewed as learning opportunities rather than failures has been shown to be particularly important in emergency contexts (Lindström and Falk 2023). Psychological safety supports open interpersonal communication, encourages reflective practice and enhances team performance under pressure, particularly when members are unfamiliar with one another. From an ethical perspective, the International Council of Nurses (ICN 2021) emphasises the responsibility of nurses to advocate for a working environment that promotes safety, respect and continuous learning. This ethical mandate reinforces the importance of fostering a psychologically safe climate in healthcare settings.

Research further shows that psychological safety is associated with greater engagement in quality improvement initiatives, meaningful contributions in interprofessional settings and stronger commitment to patient advocacy (Feng et al. 2020; Montgomery et al. 2025). Leadership behaviours characterised by openness, inclusiveness and support are well‐established enablers of psychological safety in clinical practice, fostering environments where healthcare professionals feel safe to speak up, learn and collaborate (Ajjawi et al. 2022). Simulation‐based trauma team training has demonstrated significant improvements in clinicians' confidence, leadership skills and non‐technical skills critical for effective teamwork in trauma care (Murphy et al. 2019).

Despite the growing body of literature on psychological safety in healthcare, relatively little is known about the subjective experiences of specialist nurses working in ad hoc teams during emergency care. O'Donovan and McAuliffe (2020) suggest that factors such as team familiarity, leadership inclusiveness and organisational support shape psychological safety perceptions in healthcare professionals. This aligns with broader evidence identifying leadership behaviour, organisational culture and team climate as key determinants of psychological safety (Purdy et al. 2023; El‐Ashry et al. 2025).

Addressing this knowledge gap is essential, as nurses' perceptions of psychological safety directly influence their willingness to speak up, report concerns and engage in patient safety practices. By exploring these perceptions, healthcare organisations can design targeted strategies to strengthen psychological safety, thereby improving teamwork, clinical decision‐making and patient outcomes in emergency care settings.

## 3 The Study

### 3.1 Aim

The aim of this study was to describe nurses' experiences of psychological safety during emergency care in ad hoc teams.

## 4 Methodology

### 4.1 Design

An interpretive‐descriptive qualitative design was employed as the overarching methodological approach to analyse the study's findings in relation to the professional role (Thorne et al. 1997). This design is grounded in the understanding that human experiences are shaped by complex social interactions and contextual factors (Thompson Burdine et al. 2021). By utilising an interpretive‐descriptive approach, the study facilitated a nuanced understanding of specialist nurses' experiences, recognising that each individual's perspective is unique and influenced by social dynamics (Thorne et al. 1997).

### 4.2 Theoretical Framework

This study is grounded in Edmondson's theory of psychological safety, which conceptualises psychological safety as an emergent property of interactions (Edmondson 1999). This perspective contrasts with approaches that attribute psychological safety to individual dispositions or to overarching organisational culture (Edmondson and Lei 2014). Edmondson's framework emphasises behaviours such as speaking up, admitting errors and questioning established practices—actions that entail interpersonal risk and can only thrive when team members feel assured that they will not face embarrassment, rejection or punishment (Edmondson 1999).

Developed through empirical research in hospital teams (Edmondson 1999), Edmondson's theory provides a pertinent analytical lens for exploring the situated, relational and dynamic dimensions of psychological safety in hospital acute settings.

### 4.3 Sample and Participants

Purposive sampling was used to recruit experienced nurses with a background in ad hoc teamwork during emergency care. In Sweden, these are specialist nurses who, in addition to being registered nurses, have completed 1 year of advanced specialist training resulting in a master's degree. Inclusion criteria: eligible participants were specialised in emergency care, intensive care or anaesthesia, and had at least 1 year of experience in their current role. Exclusion criteria: nurses without formal specialist nursing training, specialist nurses with less than 1 year of experience in emergency care, and specialist nurses with no recent experience of ad hoc teamwork in acute situations. This ensured that participants had taken part in multiple emergency situations in ad hoc teams and possessed sufficient expertise to provide rich accounts during interviews.

Participants from two hospitals were invited based on their relevant experience and capacity to provide rich, detailed insights aligned with the research aim. An email invitation was sent to 15 specialist nurses, and eligibility was verified against the inclusion criteria for those who responded. The initial sample consisted of six nurses; an additional three were recruited via contacts between colleagues through snowball sampling, resulting in a final sample of nine. All participants gave informed consent and were fully aware that participation was voluntary and that they could withdraw from the study at any point without consequence.

The concept of information power guided the determination of sample size (Malterud et al. 2016). Assessment regarding the specificity of the study, consecutive assessment of the quality of the interviews, and the richness of the data led to the recruitment process ending after nine interviews. A focused research purpose, strong interviewer–participant dialogue, and a conceptually informed approach enabled the collection of nuanced data without redundancy. These steps ensured the sample was adequate to support analytical depth and relevance, in line with COREQ guidelines for transparency and rigour in qualitative research.

### 4.4 Data Collection

All interviews were conducted by the first author, a specialist nurse with over 8 years of clinical experience. Telephone interviews were held with this purposive sample of specialist nurses (*n* = 9). An interview guide was developed to ensure that questions were relevant to the study aim and explored nurses' experiences of psychological safety during emergency care in ad hoc teams. The formulation of several questions was informed by Edmondson's conceptualisation of psychological safety. To refine the guide and enhance its clarity and relevance, a pilot interview was conducted.

During the interviews, participants were asked to describe what psychological safety meant to them, how they experienced it in emergency care situations, and how it influenced their work within ad hoc teams. The interviews were conducted via telephone in a one‐on‐one setting between the interviewer and each participant, with no other individuals present. Follow‐up questions were used to probe more deeply into participants' responses. Data were collected between May and June 2024, with interviews lasting between 27 and 47 min (*M* = 35 min). All interviews were audio‐recorded, transcribed verbatim, checked for accuracy and anonymised by removing personal identifiers from the transcripts. The transcripts were not returned to participants due to consideration of confidentiality and to minimise the participants' burden, while ensuring data accuracy through thorough interview techniques, detailed field notes and researchers' reflexivity. No follow‐up interviews were necessary as the initial interview provided comprehensive and rich data sufficient for the study's analytical needs. Field notes were made during and immediately after each interview to capture contextual details and the interviewer's reflections, enhancing data richness and reflexivity. The field notes were securely stored accessible only to the first author. Audio recordings are stored on a secure server at Örebro University accessible only to the primary researchers to secure confidentiality. The recordings will be erased 5 years after the scientific publication of the findings. Anonymised transcripts were available only to the two researchers and were securely maintained in the research process.

### 4.5 Data Analysis

Reflexive thematic analysis, as described by Braun and Clarke (2022), was employed to analyse the collected data. This six‐phase method was chosen for its theoretical flexibility and its ability to identify meaningful patterns within participants' experiences in relation to the study's aim (Braun and Clarke 2019; Nowell et al. 2017).

The analytic process began with the first author (JS) transcribing and repeatedly reading all interviews to gain deep familiarity with the data. Initial reflections and analytic notes were documented during this stage. An inductive coding approach was applied, consistent with the reflexive stance of thematic analysis, emphasising the researcher's active, interpretive role in making sense of meanings within the data (Braun and Clarke 2019).

Subsequently, codes were grouped to form initial themes, which were iteratively reviewed for coherence and relevance against both the coded extracts and the entire datasets. This cyclical process of refinement, including merging or redefining themes aligns with quality criteria for reflexive analysis as outlined by Morris (Morriss 2024). Table 1 illustrates an example of this analytic process.

**TABLE 1:** Example of illustrative data from reflexive thematic analysis—*Theme: Interpersonal skills: Implications of psychological safety*.

Code	Illustrative quotations	Preliminary interpretation	Thematic interpretation
Trust in colleagues' competence	“I usually enter the room trusting that people know their job.”	“I think I make a quick assessment of the person in front of me.”	Trust is positioned as a default orientation, yet remains conditional upon observed behaviour.

Understanding participants' meanings was achieved through rigorous qualitative analytic techniques emphasising researcher reflexivity and immersion in the data. Although transcripts were not returned for member checking, data accuracy and interpretation validity were ensured by triangulating detailed field notes with audio recordings and transcripts, following transparency and trustworthiness principles (Nowell et al. 2017). The researcher engaged deeply with the data, continuously reflecting on analytic decisions to capture the nuanced meanings participants attributed to their experiences (Braun and Clarke 2021). Once finalised, the themes were clearly defined and named to reflect their core analytic essence and connection to the research aim, consistent with the interpretative orientation of Braun and Clarke (2021) approach to reflexive thematic analysis.

### 4.6 Reflexivity

Reflexivity was integral to this study. Given the use of reflexive thematic analysis, the researchers' backgrounds and preunderstandings were explicitly acknowledged and critically examined throughout the analytic process. Reflexivity involved continuous reflection on how individual perspectives shaped the generation and interpretation of meaning, thereby enriching the analysis and deepening contextual understanding of the phenomenon. The first author's professional background as a specialist nurse in anaesthesia and emergency care provided valuable insider insight, facilitating nuanced data interpretation while simultaneously carrying the risk of bias. To mitigate such influence, reflexive journaling and ongoing critical dialogue were employed. In contrast, the last author's expertise in health professions education contributed a critical outsider perspective, which enhanced analytic rigour and challenged taken‐for‐granted assumptions. The interplay between insider and outsider positions not only reinforced the credibility of the findings but also aligned with the principles of reflexive thematic analysis, resulting in a nuanced and trustworthy interpretation of the data.

### 4.7 Ethical Considerations

This research project was approved by the Swedish Ethical Review Authority (No. 202401397‐01). The participation was voluntary and was assessed for not having any negative consequences for either employment or personal life. Every participant was presented with both written and verbal information about the study; the participant was ensured their confidentiality and the right to withdraw from the study at any time during the process, without negative consequences. Prior to the data collection the written consent was obtained. None of the participants received financial or other benefits for participating.

### 4.8 Rigour and Quality

This study followed the Eight Big‐Tent criteria for qualitative quality (Tracy 2010) to ensure rigour and trustworthiness. Rigour was maintained using reflexive thematic analysis, with detailed documentation of each analytical phase. Sincerity was upheld by ongoing reflexivity and transparent discussion of the researchers' preconceptions, particularly their clinical backgrounds in emergency care. Credibility was strengthened through meticulous transcription and repeated verification of recordings, complemented by a transparent and comprehensive audit trail detailing all methodological decisions. The study aimed to offer resonance through descriptions that capture the complexities of team dynamics and psychological safety in ad hoc emergency teams. Its significant contribution lies in advancing understanding of factors influencing nurse confidence and collaboration under pressure. Ethical principles were strictly followed, ensuring ethics through informed consent and participant confidentiality. Finally, the study demonstrates meaningful coherence by aligning research aims, methodology, analysis and conclusions to illuminate psychological safety in emergency care ad hoc teams.

## 5 Findings

Nine specialist nurses in intensive care, emergency care or anaesthesia participated in the study. The majority were female (*n* = 7). Three nurses worked in the thoracic intensive care unit, three in trauma emergencies, two in the paediatric intensive care unit, and one in the operating unit. Their experience of working in ad hoc teams during emergency care ranged from 2 to 22 years.

The analysis resulted in four principal themes concerning psychological safety in emergency care contexts involving ad hoc teams. These themes, along with representative participant quotes, are summarised in Table 2.

**TABLE 2:** Key findings.

Theme	Key findings	Illustrative quotations
5.1 Interpersonal skills: Implications for psychological safety	Empathy, clear communication, responsibility, stress tolerance and cooperation were central to fostering psychological safety. Personal maturity, humility, respectful communication and mindful use of tone and body language supported a safe climate. Stress and time pressure could undermine safety.	*“It's clear that you become a little unsure if it's… if it's someone who is unpleasant or if you get snarky answers if you ask something…” (B8)* *“With experience, I've developed greater confidence—not because I know everything better, but because I know what I don't know and trust that we do our best.”* (B2)
5.2 Individuality and team dynamics	Lack of prior relationships and unclear understanding of team members' competence created uncertainty. Familiarity and mutual trust improved safety. Negative past experiences and strong personalities could reduce openness. Role clarity early in collaboration was important.	*“If you don't know the person next to you… then we don't know what to expect from each other…”* (B2) *“I'd rather go to someone I am familiar with and comfortable with… and know what they can and can't do…” (B4)*
5.3 Confidence, competence and collaboration	Balance between self‐reliance and willingness to seek help enhanced performance. Clinical competence and situational awareness improved decision‐making and safety. Unfamiliar situations, critical questioning and negative attitudes could erode safety. Less experienced nurses were more vulnerable.	*“You have to have personal maturity and knowledge about yourself and how you react…”* (B4) *“You look for mistakes instead of helping people find the right ones…”* (B9)
5.4 Organisational responsibility for promoting psychological safety	Visible, engaged leadership and constructive feedback were key to a supportive climate. Ongoing training and clear guidelines improved safety, though flexibility was required for unpredictable situations. A culture of openness and learning strengthened safety.	*“We have some training… but not enough.”* (B2) *“…there won't be sudden psychological safety in a completely dysfunctional department just because it's an emergency…”* (B2)

### 5.1 Interpersonal Skills: Implications for Psychological Safety

Specialist nurses identified interpersonal skills—such as empathy, clear communication, responsibility, stress tolerance and cooperation—as key factors in fostering psychological safety within ad hoc emergency care teams. Attitudes and demeanour were described as equally crucial, with an open and flexible approach considered essential for creating a safe environment. When these qualities were present, teams were perceived as adaptable and capable of cooperating regardless of hierarchy or preconceptions.

Body language, communication… what is said… the tone of voice… means a lot… it could make a difference. (B1)

A positive attitude—including the willingness to take initiative, make decisions and support others—was reported to contribute the effective teamwork. In contrast, negative experiences within the team undermined psychological safety and could instead be experienced as reducing confidence in one's competence and knowledge.

It's clear that you become a little unsure if it's… if it's someone who is unpleasant or if you get snarky answers if you ask something… (B8)

Several participants highlighted the role of personal maturity—defined as the capacity to manage stress, navigate complex tasks and address interpersonal challenges with responsibility and ethical awareness—together with humility, irrespective of professional background. Self‐awareness, emotional regulation and maintaining professional conduct, including acknowledging and seeking support when needed, were considered essential.

With experience, I've developed greater confidence – not because I know everything better, but because I know what I don't and trust that we do our best. (B2)

Respectful communication and active listening were identified as integral components of personal maturity; their absence was perceived to diminish psychological safety. Working under pressure in ad hoc teams required not only resilience, maturity and flexibility but also conscious use of tone and body language, as these significantly influenced the teams sense of safety.

Because it's enough for someone to step in and have a bad day… a worse tone… and grumble a bit. That can ruin the mood for the whole team… That can upset the roundest of circles… (B7)

Frustration or anger in response to stress could easily and negatively affect the entire team. Maintaining calm and focus was therefore deemed vital for both effectiveness and safety.

### 5.2 Individuality and Team Dynamics

Participants noted that individual strengths, weaknesses and experiences inevitably shaped overall team dynamics. Psychological safety was closely linked to the influence of personality and familiarity.

It's very different… It can go smoothly, or it can come to a complete stop. The personalities in the team decide that. (B6)

A primary challenge was the absence of prior working relationships or familiarity, both within and across professions, which generated uncertainty about mutual expectations. This lack of familiarity heightened uncertainty—especially when combined with varying competence levels—amplifying the need for trust in each other's expertise and clarity about professionals' roles.

If you don't know the person next to you and they don't know me, then we don't know what to expect from each other…. (B2)

Conversely, previous collaboration fostered familiarity that strengthened psychological safety by creating a sense of belonging and trust.

I'd rather go to someone I am familiar with and comfortable with… and know what they can and can't do… (B4)

However, past negative experiences with disrespectful colleagues damaged trust, increasing anxieties about mistakes despite recognition of competence and skill. Participants emphasised kindness and openness as key to maintaining safety and willingness to learn.

Leadership also played a decisive role. Leaders who acknowledged their own uncertainties created environments where others felt safe to do the same, while unclear or inexperienced leadership undermined confidence.

Unclear leadership, lack of experience and treatment from others in the team affect psychological safety… I would say. (B1)

A calm and composed leadership presence—particularly when clearly assuming roles during acute situations—was repeatedly described as critical.

If someone steps forward and is clear and calm… then… colleagues who are worried and stressed feel…okay, now it seems under control. (B5)

Role clarity at the start of collaboration in ad hoc teams reduced ambiguity, fostered confidence and promoted open communication. These conditions enabled a culture of trust, where team members felt safe to admit uncertainty and contribute collaboratively to problem‐solving—essential for maintaining psychological safety in dynamic ad hoc teams.

### 5.3 Confidence, Competence and Collaboration

The importance of balancing self‐reliance with willingness to seek assistance was emphasised. Experience in managing complex, dynamic situations was seen to offer a broader perspective, enabling rapid prioritisation and safeguarding of patient safety. Clinical competence—spanning expertise, critical thinking and effective communication—enhanced psychological safety by increasing decision‐making confidence and the ability to navigate high‐pressure environments.

You have to have personal maturity and knowledge about yourself and how you react… (B4)

Mutual trust in team members ‘clinical capabilities was regarded as a key process to managing complex emergency cases calmly and effectively. Rather than functioning merely as a prerequisite for teamwork, this facilitated open knowledge‐sharing, promoted psychological safety and enabled team members to flexibly shift between leading and deferring roles as situations demanded. This was experienced to foster rapid coordination and collective problem‐solving, strengthening the team's resilience and overall capacity.

Nurses' explicit recognition of their own strengths and limitations was fundamental for effective delegation and optimal use of resources. Their competence in self‐assessment enabled the participants to take informed, situationally appropriate decisions about task aligned with professional roles.

I usually take my place… not right at the patient's side but at the foot of the bed… I just pick up on what needs to be done and delegate… (B2)

In turn, this process facilitated a more comprehensive situational awareness, as individual expertise was harnessed and distributed according to context‐specific demands. Such reflective decision‐making not only supported efficient workflow but also promoted collective responsibility and enhanced overall team functioning by ensuring that clinical tasks were matched with the appropriate level of competence.

No one will know everything… you kind of have… much more understanding and respect for… You must be kind and pleasant… that's important… (B7)

Despite experience, unfamiliar or novel situations induced uncertainty and stress. Even familiar challenges, under intense pressure, could erode safety. Being questioned by colleagues could amplify this. Direct criticism was generally perceived as diminishing both self‐confidence and a sense of psychological safety. Among leaders operating in high‐pressure contexts, such experiences not only triggered moments of uncertainty but could also give rise to frustration, thereby influencing how they navigated decision‐making and interactions with their teams.

You look for mistakes instead of helping people find the right ones… (B9)

Less experienced nurses reported a heightened sense of insecurity, often linked to concerns about making mistakes or not fully meeting the expectations placed upon them. This insecurity was described as influencing how they approached tasks, interacted with colleagues and managed responsibility in their daily work. Negative team attitudes were felt to weaken autonomy and decision‐making, ultimately undermining safety—particularly for less experienced nurses.

### 5.4 Organisational Responsibility for Promoting Psychological Safety

Participants stressed the pivotal role of organisational and unit managers in maintaining psychological safety. A lack of active leadership was viewed as detrimental, while visible, engaged leaders who offered constructive feedback and encouraged learning were considered vital.

We have some training… but not enough. (B2)

Insufficient ongoing training was described as a barrier that limited opportunities to maintain and develop competence. Several participants expressed that the lack of structured learning left them feeling unprepared for new or complex situations, which in turn created a sense of uncertainty and affected confidence in their professional role.

Is it okay to not know everything? … To admit that you are unsure or feel insecure? (B5)

Failure by managers or organisations to acknowledge their role in shaping safety experiences was seen as compromising psychological safety. Participants emphasised the need for clear, updated guidelines, while accepting that emergencies could never be completely standardised.

…I think psychological safety is a fresh commodity… a lot comes with knowledge and learning… to study further… (B6)

They underlined that, beyond formal training, individual preparation, such as scenario familiarisation was key. Departmental climates needed to be inviting, permissive and supportive, set by leadership but maintained collectively. Such environments encouraged openness and reduced hesitation to seek help, which participants described as crucial in fast‐changing situations. When these conditions were absent, however, participants reported feelings of vulnerability and reluctance to contribute, which hindered both collaboration and confidence.

…there won't be sudden psychological safety in a completely dysfunctional department just because it's an emergency… (B2)

An organisational culture of openness, where mistakes were reframed as learning opportunities, was deemed essential. Leaders who prioritised well‐being, fostered cohesion and actively reduced stress and uncertainty were perceived as creating a stable foundation for meeting the demands of specialised emergency care.

## 6 Discussion

**FIGURE 1:**
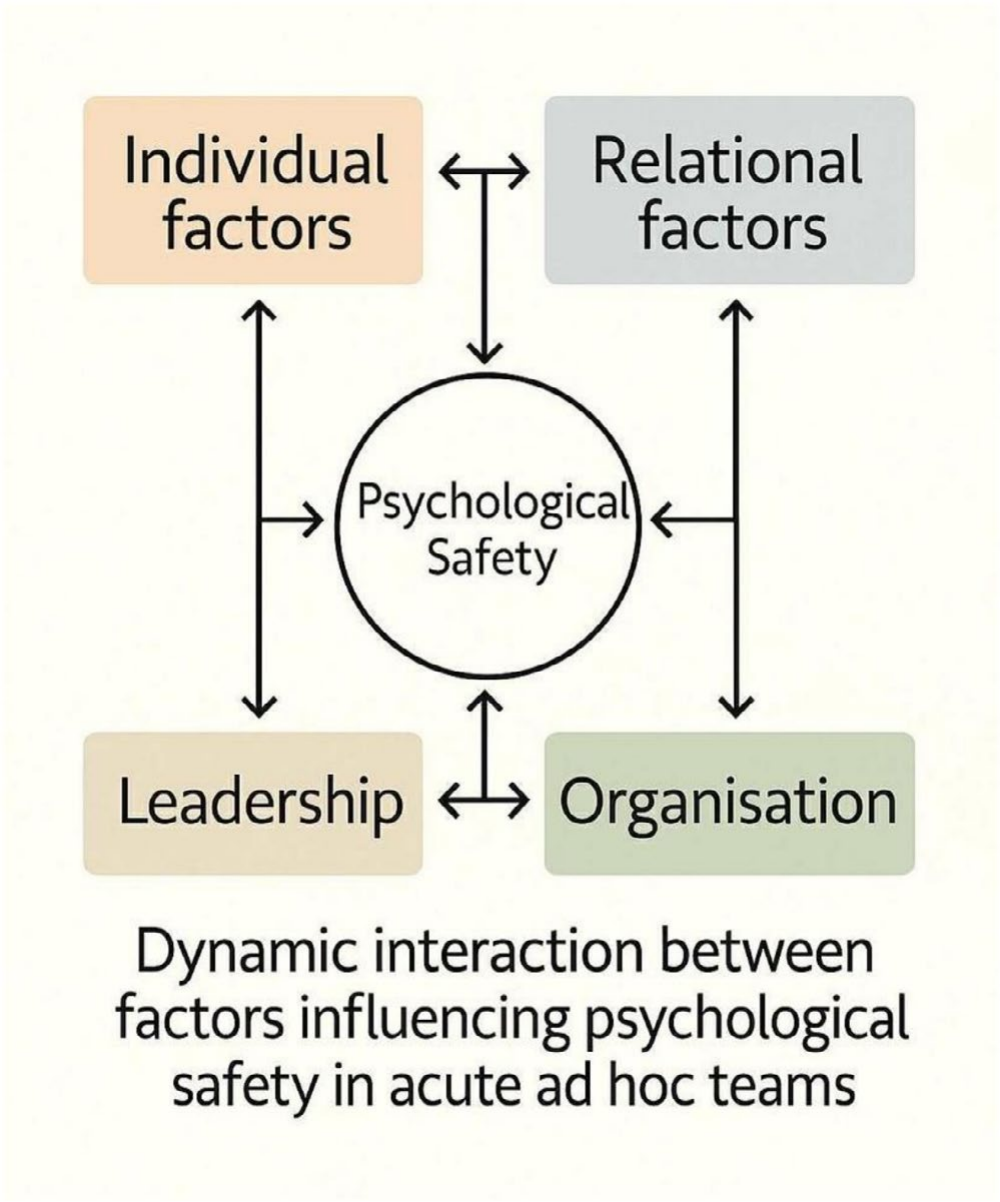
The dynamic interplay between individual, relational, leadership and organisational factors influencing psychological safety in acute ad hoc emergency teams.

This study demonstrates that psychological safety among specialist nurses in ad hoc teams within emergency care is a dynamic, multifaceted and context‐dependent phenomenon. It is continuously shaped by the interplay of individual attributes, relational processes, leadership and organisational culture. The findings affirm and extend Edmondson's (1999) model by illustrating how these dimensions interact, particularly in high‐pressure environments where team continuity is limited. Edmondson draws a conceptual distinction between psychological safety and group cohesiveness; psychological safety enables open dissent and learning, whereas cohesiveness may suppress constructive challenge and reinforce conformity. The team becomes, in this view, the metaphorical crucible in which psychological safety is collectively created, maintained or undermined. However, it is important to recognise that these factors are not easily distinguishable but rather intricately intertwined in a complex and dynamic web. Individuals' emotional maturity and interpersonal skills influence the development of relationships and communication, which are in turn shaped and supported by leadership and organisational culture. These dimensions continuously reinforce or undermine each other, collectively creating the team's psychological climate. The study findings indicate that participants experienced psychological safety as a dynamic and integrated system, in which individual, relational, leadership and organisational factors intertwined fluidly, making the whole perceived as greater than the sum of its parts. Figure 1 underscores the complexity and dynamic interdependence of factors influencing psychological safety, especially in the context of acute ad hoc teams such as those found in emergency healthcare settings. Rather than being a static or individual characteristic, psychological safety arises through ongoing interactions among personal, relational, leadership and organisational dimensions. This perspective emphasises the need for multifaceted strategies and adaptive intervention to cultivate and sustain psychological safety in high‐pressure clinical environments (Edmondson 1999, 2019).

The findings underscore the central role of interpersonal skills—such as empathy, respectful communication, emotional regulation and professional demeanour—in maintaining psychological safety in high‐pressure emergency care settings which aligns with the critical importance of non‐technical skills alongside technical expertise in healthcare teamwork for safe care delivery (Han and Roh 2020). Edmondson's standpoint that psychological safety is a fragile, dynamic state, easily enhanced by constructive interactions but threatened by negative tones, dismissive attitudes or non‐verbal cues still holds in view of our findings (Edmondson 1999).

The dual role of stress was evident in the findings. Personal maturity, humility and balanced self‐confidence emerged as key protective factors, buffering stress and enabling trust and cooperation in ad hoc teams. Conversely, arrogance and lack of self‐awareness undermined collaboration and amplified stress contagion among team members. Such findings extend Edmondson's framework by showing that deliberate emotional regulation and reflective practice can stabilise team climate even amidst hierarchical complexities and unfamiliar team compositions (Purdy et al. 2023). Our study also challenges traditional assumptions about hierarchy in emergency care: interpersonal skills and mutual respect often outweighed positional authority in fostering psychological safety. This supports O'Donovan and McAuliffe (2020) findings that psychological safety is co‐created by leaders and peers through trust and open communication that can flatten hierarchical barriers. The dual nature of stress manifested distincly—moderate levels of stress enhanced cognitive focus, whereas excessive or poorly regulated stress impaired performance and compromised relational safety. These results call for systemic stress‐management strategies in emergency settings, including simulation‐based training, structured debriefing, peer support and greater awareness of how interpersonal behaviour shapes team dynamics. Evidence shows that targeted stress management, such as mindfulness and cognitive‐behavioural techniques, can reduce burnout and build resilience in healthcare teams (Bartone et al. 2022; Jiang et al. 2025).

Our findings resonate with recent research emphasising that psychological safety in emergency care settings is profoundly influenced by trust, clear role definition and prior relational familiarity across professions. Studies indicate that uncertainty stemming from unfamiliarity and varying competence levels can significantly undermine team cohesion and overall safety (Granroth Schulman and Wentin 2025). This aligns with findings that healthcare staff's feelings of psychological safety are closely linked to their sense of physical safety, access to support and stable team compositions, especially in high‐risk, fast‐paced environments such as acute mental health and emergency care (Vogt et al. 2024).

Respectful communication and openness to seeking help emerged as essential elements, consistent with evidence that leadership behaviours which acknowledge uncertainty foster a climate that encourages speaking up and reduces fear of negative consequences (Purdy et al. 2023; Mehta et al. 2024). Challenges posed by strong personalities and overconfidence reflected literature showing that team composition critically influences whether members in ad hoc teams feel safe to contribute under pressure (Purdy et al. 2023). Psychological safety, as Edmondson (1999, 2019) underscores, is not a fixed trait but a dynamic interpersonal state, continuously evolving through interaction and reliant on deliberate cultivation via team building, interpersonal skill development and respect‐based leadership. Consequently, comprehensive strategies such as equitable workload distribution, clear communication, simulation training, structured briefing and peer support systems are indispensable for sustaining collaboration under the exceptional pressures of acute care (Bartone et al. 2022; Jiang et al. 2025).

In line with this, participants in our study emphasised the importance of balancing self‐reliance with willingness to seek assistance, alongside clinical competence—encompassing expertise, critical thinking and communication. This combination enhanced confidence in decision‐making under pressure. Yet our findings also reveal that even experienced nurses can have their psychological safety undermined in novel or challenging situations, particularly when their competence or decisions are questioned. Less experienced nurses described feeling especially vulnerable, echoing recent scholarship highlighting the uneven distribution of voice and influence in time‐critical teams (Tham et al. 2025). Recent studies also show that psychological safety functions both as an individual state shaped by personal skills, emotional maturity and reflective practice, and as a collective resource reliant on mutual trust and supportive team behaviours (Montgomery et al. 2025). Inclusive and authentic leadership is critical for fostering psychological safety by encouraging open communication and reducing absenteeism among nursing staff, boosting performance and well‐being (Hessler et al. 2024; Shankar et al. 2025). Moreover, supportive leadership styles significantly contribute to nurses' intention to remain in their positions and provide safe, patient‐centred care (Cho et al. 2023).

Finally, participants consistently identified leadership and organisational culture as critical to psychological safety. Visible, engaged leaders who modelled inclusivity, offered constructive feedback, and fostered open dialogue were seen to reduce fear and encourage speaking up, consistent with foundational frameworks by Edmondson (2019) and findings from Han and Roh (2020). Recent research further highlights that health‐promoting leadership styles, characterised by empathy, open communication and support for professional growth, significantly enhance nurses' psychological safety and reduce withdrawal behaviours (Esmaeilbeigi et al. 2025). Inclusive leadership promotes a culture where diversity of thought is valued, and staff feel safe to express concerns without fear of retribution, thereby fostering engagement and commitment (Hirvikallio et al. 2024). Conversely, absent or inconsistent leadership, lack of ongoing training and unclear or outdated protocols were noted to undermine team functioning and psychological safety. Organisational cultures that legitimise uncertainty and treat mistakes as learning opportunities contribute to a resilient workforce, enabling rapid trust building and cohesion in high‐pressure ad hoc teams (Esmaeilbeigi et al. 2025). Our findings extend this by illustrating how the departmental climate—formed through interactions between management and team members—can either facilitate or hinder the swift development of trust and cohesion critical in emergency care settings. Taken together, these insights underscore that the dynamic nature of ad hoc emergency care teams requires a model that integrates individual and system‐level factors to advance both theory and practice of psychological safety.

### 6.1 Strengths and Limitations

Most participants were recruited from a large hospital, with only one participant representing a medium‐sized hospital, which constitutes a limitation of the study. However, a notable strength was that the participating experienced nurses were active in a variety of clinical activities, thereby ensuring that the emergency care context encompassed diverse patient groups and varying constellations of ad hoc teams.

### 6.2 Recommendations for Further Research

The present study highlights that psychological safety is essential in ad hoc teams, where members often work together for the first time in time‐limited emergency situations. Many participants described that clear structures, open communication and mutual respect during emergency care improved their sense of psychological safety despite unfamiliar contexts. Further research should examine how psychological safety develops dynamically within ad hoc teams, and identify strategies that leaders and team members can use to rapidly foster trust and openness among previously unacquainted participants. Investigating interventions that strengthen psychological safety in ad hoc teams, as well as exploring the effects on collaboration, learning and outcomes in such settings is warranted.

### 6.3 Implications for Policy and Practice

The results suggest several priorities for healthcare organisations and policymakers. Leadership development should focus on inclusive leadership and communication, with clinical training extended to cover conflict management and stress regulation. Structured opportunities for relationship‐building, even in ad hoc or short‐term teams are essential to foster trust and cohesion. Embedding psychological safety in organisational culture can enhance staff well‐being, improve collaboration and result in better patient outcomes.

## 7 Conclusions

This study demonstrates that psychological safety in ad hoc emergency care teams is a fragile, multifaceted and dynamic construct shaped by interpersonal skills, leadership and organisational culture. Sustained efforts to create supportive environments—marked by open communication, mutual respect and proactive leadership—enable specialist nurses to collaborate confidently, even under acute stress. The findings extend current models influenced by Edmondson's pioneering work by showing how individual, relational and structural factors intricately interact to shape the team climate, emphasising that psychological safety is best understood as a living system where the boundaries between influencing factors are fluid. Ultimately, targeted strategies that strengthen interpersonal conduct, foster inclusive and supportive leadership and embed psychological safety in organisational culture are essential for promoting effective teamwork and optimal patient safety in high‐pressure care settings.

## Author Contributions

Made substantial contributions to conception and design, or acquisition of data, or analysis and interpretation of data: J.S., S.E. Involved in drafting the manuscript or revising it critically for important intellectual content: J.S., S.E. Given final approval of the version to be published. Each author should have participated sufficiently in the work to take public responsibility for appropriate portions of the content: J.S., S.E. Agreed to be accountable for all aspects of the work in ensuring that questions related to the accuracy or integrity of any part of the work are appropriately investigated and resolved: J.S., S.E.

## Funding

This research project was approved by the Swedish Ethical Review Authority (No. 202401397‐01).

## Conflicts of Interest

The authors declare no conflicts of interest.

## Data Availability

No other paper use the same dataset.
